# Systematic Review of Functional MRI Applications for Psychiatric Disease Subtyping

**DOI:** 10.3389/fpsyt.2021.665536

**Published:** 2021-10-22

**Authors:** Lucas Miranda, Riya Paul, Benno Pütz, Nikolaos Koutsouleris, Bertram Müller-Myhsok

**Affiliations:** ^1^Department of Statistical Genetics, Max Planck Institute of Psychiatry, Munich, Germany; ^2^Department of Precision Psychiatry, Max Planck Institute of Psychiatry, Munich, Germany; ^3^Department of Psychiatry and Psychotherapy, Section for Neurodiagnostic Applications, Ludwig-Maximilian University, Munich, Germany; ^4^Department of Health Data Science, Institute of Translational Medicine, University of Liverpool, Liverpool, United Kingdom

**Keywords:** functional MRI (fMRI), personalized medicine, disease subtyping, biotypes, machine learning, unsupervised learning, clustering, translational psychiatry

## Abstract

**Background:** Psychiatric disorders have been historically classified using symptom information alone. Recently, there has been a dramatic increase in research interest not only in identifying the mechanisms underlying defined pathologies but also in redefining their etiology. This is particularly relevant for the field of personalized medicine, which searches for data-driven approaches to improve diagnosis, prognosis, and treatment selection for individual patients.

**Methods:** This review aims to provide a high-level overview of the rapidly growing field of functional magnetic resonance imaging (fMRI) from the perspective of unsupervised machine learning applications for disease subtyping. Following the PRISMA guidelines for protocol reproducibility, we searched the PubMed database for articles describing functional MRI applications used to obtain, interpret, or validate psychiatric disease subtypes. We also employed the active learning framework ASReview to prioritize publications in a machine learning-guided way.

**Results:** From the 20 studies that met the inclusion criteria, five used functional MRI data to interpret symptom-derived disease clusters, four used it to interpret clusters derived from biomarker data other than fMRI itself, and 11 applied clustering techniques involving fMRI directly. Major depression disorder and schizophrenia were the two most frequently studied pathologies (35% and 30% of the retrieved studies, respectively), followed by ADHD (15%), psychosis as a whole (10%), autism disorder (5%), and the consequences of early exposure to violence (5%).

**Conclusions:** The increased interest in personalized medicine and data-driven disease subtyping also extends to psychiatric disorders. However, to date, this subfield is at an incipient exploratory stage, and all retrieved studies were mostly proofs of principle where further validation and increased sample sizes are craved for. Whereas results for all explored diseases are inconsistent, we believe this reflects the need for concerted, multisite data collection efforts with a strong focus on measuring the generalizability of results. Finally, whereas functional MRI is the best way of measuring brain function available to date, its low signal-to-noise ratio and elevated monetary cost make it a poor clinical alternative. Even with technology progressing and costs decreasing, this might incentivize the search for more accessible, clinically ready functional proxies in the future.

## Introduction

### Psychiatric Disease Prevalence

Psychiatric disorders have a long history of being classified based solely on their associated symptoms, with the first systematic analysis attempts dating back as far as the late 1800s (1). Since the introduction of the Diagnostic Manual of Mental Disorders (DSM) back in 1952 (2), and most notably since the inclusion of operationalized criteria in 1978 in the DSM-III (3), statistics on discrete pathological entities and their combination began to accumulate, yielding the potential of understanding psychiatric epidemiology in a consistent way. The last version of the DSM manual (DSM-5), published in 2013 (4), contains 297 discrete disorders categorized into 11 broad classes, grouped by evidence of co-occurring symptoms. Current prevalence estimates indicate that, on average, more than one in six individuals (17.6%) have experienced at least one common psychiatric disorder within the last year and almost three in ten (29.2%) during their lifetime (5). In an attempt to assess both the severity of the disorders and the response after individual treatment, several standardized symptom scores have been developed, including the Hamilton Depression Rating Scale (HAM-D) for Major Depression Disorder and the Positive and Negative Syndrome Scale (PANSS) for Schizophrenia, among others.

### Heterogeneity and Alternatives to Symptom-Based Diagnosis

Symptoms and clinical information can be relatively easy to acquire, and their analysis can be useful to understand the symptom prevalence in the population and assess the effectiveness of treatment on a broad scale (6). They do not, however, necessarily reflect anything about the underlying mechanisms causing them. Furthermore, given the complexity of the genetic and environmental factors at play, the same set of symptoms can arise from different causes, while the same biological causes may lead to different symptoms or phenotypes (7, 8). This is particularly important when analyzing the response to treatment, where the outcome is challenging to predict based on the symptoms alone, and response to medication is vastly heterogeneous, being treatment-resistant variants of disease not uncommon (9). For example, current estimates indicate that about 30 and 34% of medicated patients diagnosed with depression and schizophrenia, respectively, do not respond to treatment even after trying two or more drugs (10, 11). This can be interpreted as an indication of the underlying mechanistic heterogeneity of these symptom-defined disorders. In light of this concern and with the advantage of new technologies and an increasing amount of related data, several initiatives have embarked on the quest to find data-driven mechanistic disease definitions that may aid the issue. One of the most important to date has been the Research Domain Criteria (RDoC), which was introduced by the NIH (National Institute of Health) in 2009 as a framework to guide research projects in the understanding of mental disorders from a combination of different perspectives, including not only self-reported symptoms but also genomics, circuits, and behavior, among others (12). The ideas behind these mechanistic-based classifications have the potential of expanding our knowledge of mental disorders themselves, advancing biomarker discovery, and helping improve prognosis prediction and identify the best treatments for individual patients whose overlapping symptoms have distinct etiological causes, in a notion that is very much in line with those of personalized medicine (13).

### Functional MRI for Disease Subtyping

The idea of using multivariate pattern analysis to unravel the heterogeneity mentioned above and unveil subgroups of patients within already defined diseases is not new (12, 14, 15). However, the advent of massive biological related datasets (the so-called *high-throughput biology*) in areas such as genomics, transcriptomics, and proteomics, and the newly available techniques to study the brain in a non-invasive way, opened a whole new field of possibilities to study not only the underlying mechanisms of symptom-related clusters but to search for biologically defined subtypes of disease (or *biotypes*) as well. Although initial hopes were put mainly on genetics, over the years an increasing number of Genome-Wide Association Studies (GWAS) have revealed that brain disorders tend to be associated with a high number of genetic variants with tiny effect sizes (16–18). Furthermore, individual genetic alterations often overlap among symptom-defined diseases (19). While some progress in genetic biomarkers has been made using disease-specific polygenic risk scores (PRS), the usage of genetics alone for determining brain disease subtypes has been mostly elusive (20, 21). However, one of the most promising fields to pursue this aim has been neuroimaging, with Magnetic Resonance Imaging (MRI) as arguably its most proficient method to date. This technique has been increasingly used to study not only the structure of the brain (structural *MRI*) but also to measure changes in the blood oxygen levels surrounding particular regions as a proxy of neuronal activation (*BOLD fMRI*) (22). One of the most prevalent uses of this technology has been *task-based fMRI*, in which an experimental design matrix, typically convolved with a mathematical function modeling the hemodynamic response (called hemodynamic response function, or HRF), is set to explain the observed signal using a General Linear Model (GLM). While this approach has a substantial amount of literature behind it and is highly flexible due to relying on a Linear Model assumption (23), it has some notorious drawbacks. First, the most common analyses rely on *mass univariate tests*, which statistically assess differences in activation on each voxel separately, assuming independence even among contiguous regions in space. Second, it depends on an experimental task design, which, even though it can be a powerful tool for answering specific questions, is relatively hard to perform, difficult to generalize, and prone to habituation (24). An alternative that gained momentum over the last two decades has been *resting-state* fMRI, in which the subjects perform no particular task. Since it was first employed in 1995 (25), this approach allowed researchers to study the relationship between brain regions over time, which has been proven to be a useful tool to study both *functional connectivity* (Resting-State Functional Connectivity–RSFC), based on voxel correlation and yielding *undirected* connectivity networks) and *effective connectivity* (Resting-State Effective Connectivity–RSEC), based on causal modeling and yielding *directed* connectivity networks). Regardless of the analysis tool, most studies largely converged in reporting multiple robust resting-state networks across the brain, such as the primary sensorimotor network, the primary visual network, frontoparietal attention networks, and the well-studied default mode network (26). In addition, Seeley et al. proposed in 2007 the concept of *Intrinsic connectivity networks*, which refers to correlated brain regions that can be captured in either resting state or task-based neuroimaging data (27). Furthermore, recent studies interestingly show that the contribution of task performing to an individual's established connectivity networks is rather small (28), suggesting the possibility of utilizing already generated task-based fMRI data for RSFC as well (29).

The idea of the brain having stable connectivity between its different regions that can be altered in illness has been an influential hypothesis for disease subtyping. Given its potential generalizability and the robustness of the obtained results (26, 30), resting-state connectivity is currently the most used fMRI approach for both searching for and validating distinct mechanisms underlying brain disease, in an attempt to explain the aforementioned vast heterogeneity.

### Unsupervised Machine Learning on Psychiatric Disease Subtyping

Automated pattern recognition (i.e., *machine learning*) can be used to unveil subtypes in psychiatric disease in an unsupervised way (i.e., without the presence of hardcoded labels indicating for example if a disease is present or not). Given the complexity of the data at play, this set of approaches has been proven extremely useful in various settings and data domains, mainly for *clustering* and *dimensionality reduction* (13, 28). While the former deals with the process of finding subtypes in itself, the latter encapsulates a set of methods to project the data into lower-dimensional manifolds (31), in an attempt to reduce dataset size while retaining the most valuable information, which can substantially aid downstream model training.

In the case of functional MRI, unsupervised machine learning has been extensively used given the unstructured nature of the data. Its main uses include but are not restricted to parcellation of the brain into discrete functional subunits (unraveling of brain connectivity networks), the study of brain connectivity dynamics (how those networks develop over time), and grouping subjects according to their connectivity features (used for disease subtyping in itself). While the first two mentioned uses fall into the *dimensionality reduction* category, the third is inherent to *clustering*, and it will be part of the focus of this review.

Over the years, many clustering algorithms have been proposed. While a thorough classification of them is out of the scope of this review, an introductory, coarse grain subdivision of those applied in the analyzed studies, based on their general properties, can be found in Table 1.

**Table 1:** Coarse classification of clustering algorithms.

**Algorithmic family**	**Distance-based clustering**	**Graph-based clustering**	**Model-based clustering**
**Description**	A similarity/distance matrix between samples is computed, and the raw distance between samples is used for grouping similar objects together.	The similarity/distance matrix is thresholded to establish deterministic connections (edges) between samples, yielding a graph. Distance metrics at the graph level are used for determining clusters (called *communities*)	A parameterized model (typically a multimodal probability distribution) is fitted to the data. Training consists of finding the model parameters that best model the data.
**Advantages**	- Relatively low time complexity - High Computing Efficiency (they scale well to large datasets)	- Variety of highly efficient algorithms to deal with graphs (32) - Can capture the geometry of complex manifolds, a feature of interest in realistic datasets (33)	- High flexibility - Well-defined metrics for model selection (one can check how well a given model fits the data using likelihood based metrics). - Natural approach to soft clustering (probabilities are reported)
**Limitations**	- Sensitive to the selected distance metric - Number of clusters usually needs to be manually preset - Low flexibility	- Time complexity increases dramatically with the number of edges in the graph (proportional to the number of samples). - Sensitive to how the graph is constructed	- High time complexity (don't scale well for large datasets) - Flexibility comes at a cost (one must think carefully about which type of model to apply).
**Examples (mentioned across this review)**	K-means, Hierarchical Agglomerative Clustering, fuzzy c-means, Spectral clustering, Q-Factor analysis	Walktrap, Modularity maximization (Newmann's)	Gaussian Mixture Models (GMMs), Variational Bayesian GMMs

### With Great Power Comes Great Responsibility

While extremely useful when properly used, there are some inherent issues to clustering that are worth discussing before delving into the literature. For starters, clustering is in itself an ill-defined problem (34). This means that, unlike in other machine learning domains such as classification, there is neither a unique well-defined solution nor a unique definition of what a cluster is. That said, different algorithms will make different assumptions on the data that will intrinsically lead to distinct (although potentially overlapping) solutions. The choice may then rely on knowing these assumptions hold on a particular dataset, or on the empirical interpretation of a particular set of retrieved components using external variables (such as using fMRI to validate symptom or biomarker clusters, as will be presented later).

In addition, many popular clustering algorithms (although not all of them) require users to define the number of clusters they expect beforehand (typically codenamed *k*). While there are some exceptions (such as handwritten digit recognition, for example, where there are exactly 10 classes to detect), clustering is about *understanding data*, and recognizing the best number of components to define is, in most cases, a problem in itself. To solve it, researchers often rely on heuristics that compare the solutions achieved within a range of different values, exploiting a certain definition of *cluster* that the algorithm at hand uses. Besides, there is no guarantee that there are clusters at all in the data. Therefore, it is important to test the null hypothesis of *no-clusters* in our setting as well. This can be done by adding *k*=*1* to the range of values to test or using statistical methods (35).

Last but not least, there is the problem of *generalizability*, arguably one of the holy grails of machine learning as a whole. The whole point of unveiling disease subtypes from our data is to extend the results to at least a broader subset of the general population. If a solution is only valid within the boundaries of a particular study but breaks apart on different datasets, we say that the model *overfits* the data it's been trained on. To counter this issue, it is common practice to run these algorithms multiple times with different subsamplings of the dataset (by removing random sets of samples using predefined schemes, such as cross-validation, bootstrap, or Jackknife), and to assess how much the clustering solution is affected. If clusters are highly stable across samples (as measured by established metrics, such as the Adjusted Rand Index or the Jaccard Index), the solution is said to be *robust* (34, 36). While these approaches are extremely useful to test generalizability inside our data distribution, further validation steps are usually required to extend a solution to other settings, such as contrasting results to data obtained on different hospitals, or from different ethnicities [involving, for example, schemes such as leave-one-site-out cross-validation and external validation (37)]. The bottom line is: *If a subtyping study aims to draw conclusions for a certain population, generalizability to that population should be thoroughly tested*.

For a detailed review on machine learning for clinical psychiatry with a special focus on testing generalizability, please refer to (13, 38). For details on the existing unsupervised learning methods for disease subtyping, see Marquand et al. (39). For details on machine learning methods for resting-state fMRI data, refer to Khosla et al. (26).

This review will analyze the reported use to date of fMRI data for unveiling subtypes in several psychiatric disorders, and as a tool for validating subtypes reported after the analysis of other data modalities (such as symptom information, genetics, or structural MRI). The strengths and weaknesses of each approach will be discussed.

## Methods

**Figure 1:**
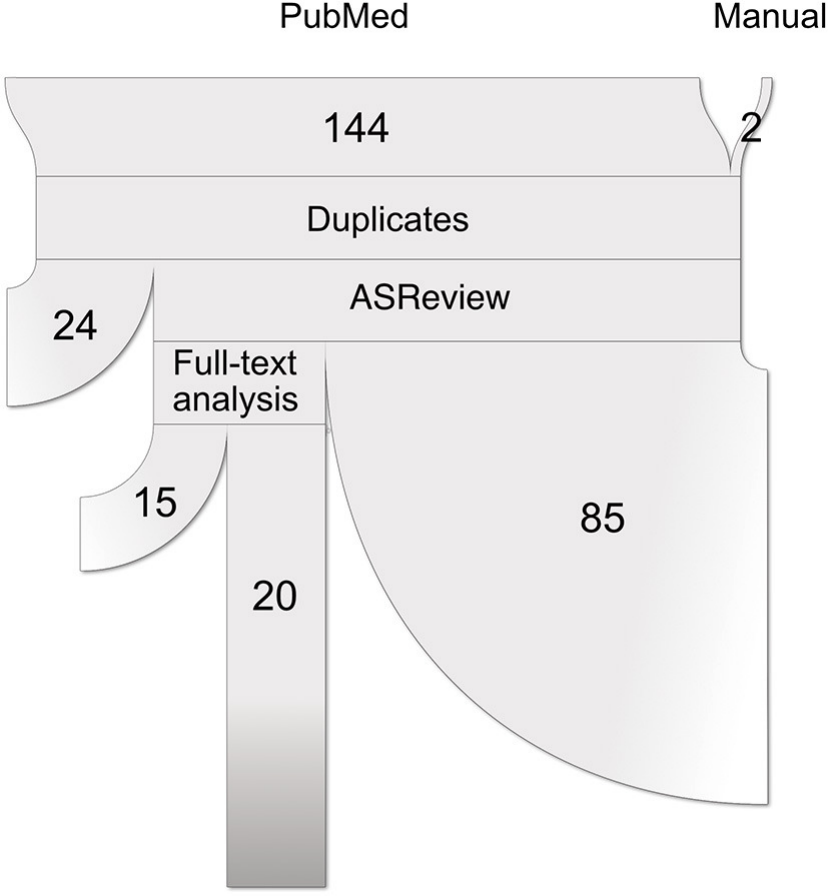
Paper selection pipeline. PRISMA flowchart (represented as a ribbon plot, which flows from top to bottom) that schematizes the employed pipeline. A total of 144 articles were retrieved from PubMed using the string “*(unsupervised learning OR clustering OR dimensionality reduction OR subtyping) AND functional MRI*”. Two articles were included after the manual search, yielding a total of 146 input studies. After several systematic filters, which included the exclusion of reviews, duplicated articles, and relevance to the defined inclusion criteria, a total of 20 articles were included in the review.

This study followed the Preferred Reporting Items for Systematic reviews and Meta-Analysis (PRISMA) statements (40). A complete flow chart of the process is shown in Figure 1. The research question intended to delve into was defined using the PICo guidelines for qualitative systematic reviews (41): *What is the state of the art in the usage of unsupervised subtyping for explaining the heterogeneity in psychiatric disease symptomatology? What role does functional MRI play in this process?*

### Search Methods for Article Retrieval

A systematic search of original articles was carried out on the PubMed database, including all non-review articles from the date of database creation up to 25 May 2020. The string “(unsupervised learning OR clustering OR dimensionality reduction OR subtyping) AND functional MRI” was entered on the search engine to retrieve all available papers in which functional MRI was used either for brain disease subtyping or for validation of brain disease subtypes obtained *via* other methods, which should include at least one of symptom information and structural MRI data.

### Article Filtering

All retrieved studies were downloaded and analyzed using PubMed metadata to filter review articles (“D016428:Journal Article”, but “D016454:Review” absent in the “*publication_types”* metadata field). The remaining studies were analyzed using the ASReview (*Automatic Systematic Reviews*) python package (42). This active-learning-based recommender system trains a classifier on the provided papers' abstracts and presents the user with the most relevant articles to review. While all abstracts included in this step were carefully studied, this tool has been proven useful for prioritization. Studies whose abstracts met the exclusion criteria (see below) were discarded. The rest was selected for full-text review.

### Inclusion/Exclusion Criteria

We retained all original non-review studies in which functional MRI was used either for brain disease subtyping directly or for validation of brain disease subtypes obtained *via* other methods, including at least one of symptom information and structural MRI data. Disease subtyping had to be carried out in an unsupervised way (no labels based on prior information except for case/controls). Precise definitions of the methods and their validation had to be included. As, given the heterogeneity of results, we think that cluster validation is currently one of the most important discussion topics in the field, articles trying to replicate or validate the results of included studies were also incorporated.

### Data Extraction for Systematic Analysis

For each article included in the final review, a set of systematically collected pieces of information was extracted and added as an entry to a table (see Tables 2–**4**). This information includes: (a) *publication year*, (b) reference, (c) *pathology*, (d) *data domain used for clustering*, (e) sample size (clustering), (f) data domain utilized for validation/interpretation, (g) sample size (validation/interpretation), (h) feature selection / dimensionality reduction algorithms utilized, (i) clustering algorithm(s) employed, (j) cluster number selection criteria, (k) robustness assessment, (l) inclusion of healthy controls at clustering time^1^ (m) testing against continuum (null hypothesis - absence of clustering structure in the data), (n) number of reported subtypes, (o) featured brain areas/networks that were recovered using fMRI.

**Table 2:** Retrieved articles in which fMRI was used to interpret symptom-based clusters.

**Publication year**	**Reference**	**Pathology**	**Data used for clustering**	**sample size (symptoms)**	**Data used for validation**	**sample size (fMRI)**	**Dimensionality reduction**	**Clustering**	**Cluster number selection**	**Robustness testing**	**Healthy Controls included**	**Testing against continuum**	**Reported subtypes**	**Featured brain areas/*networks***
2013	Taubner et al. (43)	MDD	SWAP-200 (44)	20	task fMRI (dysfunctional relationships)	20	Raw features	Q-Factor analysis (45)	variance explained (elbow method) (45)	No	No	No	2	orbitofrontal cortex, ventral striatum, temporal pole, middle frontal gyrus
2015	Geisler et al. (46)	SCZ	behavioral and cognitive scores	129	task fMRI SIRP (47)	165	PCA	K-means (36)	previous literature (48)	No	No	No	4	planum temporale, parietal operculum, precuneus cortices
2018	Dickinson et al. (49)	SCZ	PANS scores (50, 51)	549	rsfMRI	182	Raw features	GMMs (52, 53)	BIC (35)	1,000 model initializations (no left-out)	No	Yes	3	*frontoparietal working memory network*
2018	Maglanoc et al. (54)	MDD	BDI–BAI (54, 55)	1,084	rsfMRI sFC dFC	251	Raw features	GMMs (52, 53)	BIC (35)	100 model initializations (no left-out)	Yes	No	5	*default mode network, frontotemporal network*
2020	Chen et al. (56)	SCZ	PANS scores (50, 51)	1,545	rsfMRI	84	NMF (57)	fuzzy C-means (58)	fuzzy silhouette index, Xie/Beni index, partition entropy (31)	bootstrap resampling, leave-one-site-out cross- validation	No	Yes	2	ventromedial frontal cortex, temporoparietal junction, precuneus

### Characteristics of the Included Studies

**Figure 2:**
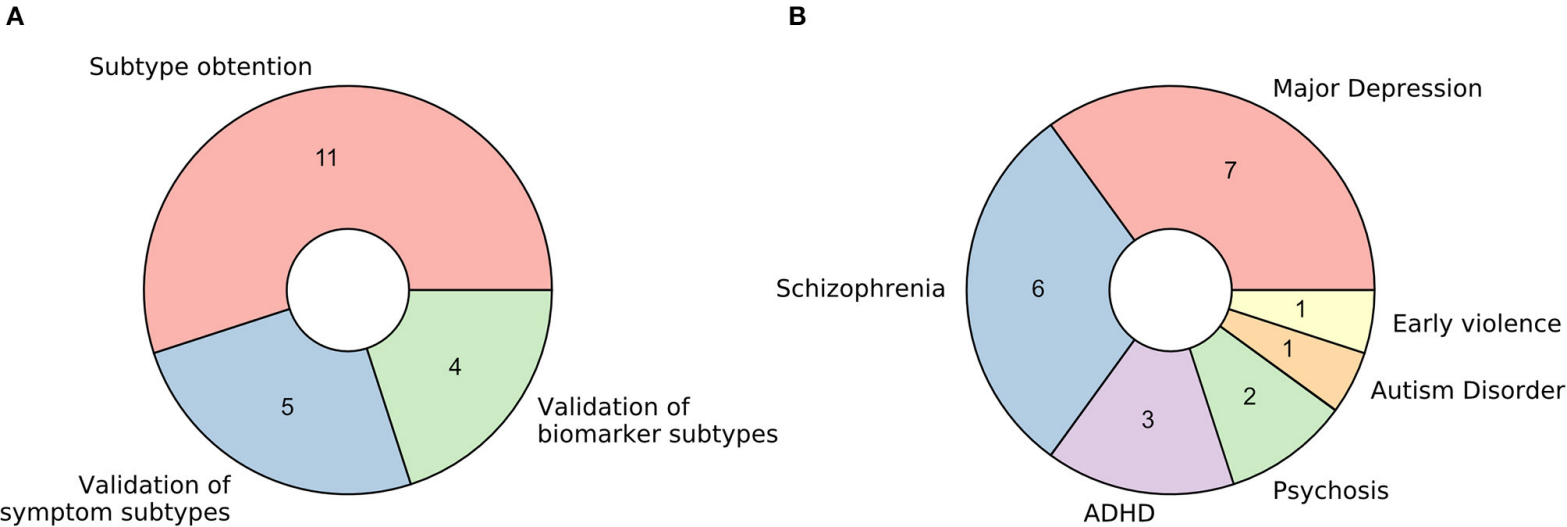
Characteristics of retrieved studies. **(A)** Donut plot representing the number of selected studies for each of the three defined categories: (a) *fMRI used for validation of subtypes obtained via unsupervised learning of symptom-related data*, (b) fMRI *used for validation of subtypes obtained via unsupervised learning of biomarkers other than fMRI (including structural MRI)*, and (c) *fMRI used for brain disease subtyping directly*. **(B)** Donut plot representing the most prevalent brain disorders that the included studies analyzed.

The 20 retrieved studies were classified into one of three categories based on the nature of the analyzed subtypes and the usage of functional MRI (Figure 2A). The classes are: (a) *fMRI used for validation of subtypes obtained via unsupervised learning of symptom-related data*, (b) *fMRI used for validation of subtypes obtained via unsupervised learning of biomarkers other than fMRI (including structural MRI)*, and (c) *fMRI used for brain disease subtyping itself*. Over the next three sections, we will analyze these three cases separately, summarizing the results that the respective studies reported and discussing the assumptions they make and the advantages and disadvantages that they imply.

Regarding the pathological entities under study, most of the articles analyzed patients diagnosed with major depression disorder and schizophrenia (35 and 30%, respectively). Psychosis, attention-deficit/hyperactivity disorder, autism disorder, and the consequences of early violence were also included (Figure 2B).

## Results

### fMRI Used for Validation of Subtypes Obtained *via* Unsupervised Learning of Symptom-Related Data

The unsupervised classification of psychiatric symptoms is not new: to our knowledge, the first papers were published back in the 1970s (12, 14, 15). The novelty of the studies presented here relies on interpreting and validating symptom clusters in terms of their underlying functional mechanisms. By comparing functional MRI data coming from patients on different clusters (or between particular clusters and healthy controls), researchers can potentially explain which mechanisms may be at play when yielding distinct sets of symptoms. The following paragraphs will explore the five papers that fall into this category.

#### Major Depression Disorder

In a pioneering study, Taubner et al. (43) addressed the symptomatic heterogeneity in a cohort of 20 patients with severe depression by clustering the personality features obtained from the Shedler-Westen Assessment Procedure (SWAP-200). As this assessment relies on clinical judgment rather than on a patient questionnaire, it is usually considered less noisy than other alternatives (44). Besides, as it is purely based on observed symptoms, it does not rely on any theoretical assumption about the mechanisms underlying depression.

In their setup, they applied a well-established method called Q-Factor analysis (45) to uncover a potential clustering structure in their data. This method aims to decompose the data matrix (of samples by features) into different components (called “factors”). The “Q” in the name indicates that factors refer to groups of individuals rather than to groups of features, as is the case in standard factor analysis. By employing an elbow method (45) on the variance explained by their factors, researchers decided to retain the two most prominent components in their data.

Furthermore, functional MRI data from an individually tailored task paradigm using dysfunctional relationship patterns were obtained from each patient whose data was used for clustering, and a whole-brain correlational analysis was done comparing the fMRI GLM parameters with the individual values extracted within the SWAP-200 factors. This way, they cataloged their two retrieved components as indicators of “Depressive personality” or “Emotional-Hostile-Externalizing Personality”, based on the analysis of the 20 SWAP-200 features that contributed the most to their partition. Moreover, the second component was linked to abnormal connectivity in the orbitofrontal cortex [strongly associated with cognitive processing and decision making (81)], the ventral striatum (a critical component of the reward system), and the temporal pole (involved in social emotion processing).

Even though they use simple, established methods, their sample size may be too low to derive reliable and generalizable conclusions. The authors call the study a hypothesis-generating experiment that might be followed up in the future. However, the recovery of previously reported, relevant activation networks seems promising.

A different approach, with a larger sample size (*n* = 1,084), was employed by Maglanoc et al. in 2018 (54). In this study, researchers used symptom data derived from Beck's depression and Beck's anxiety inventories (BDI and BAI, respectively) (55, 82), and combined individuals with and without a history of depression. These scoring systems, unlike the aforementioned SWAP-200, are self-assessed. Their reliability and competence to discriminate between subjects with and without anxiety and depression, however, has been extensively tested (83).

To cluster the symptom data, researchers applied a likelihood-based approach inspired by Gaussian Mixture Models (52, 53). One of the main advantages of this method is that it allows tuning the most suitable number of clusters in the data with a well-defined metric (the most common being the Akaike Information Criterion (AIC) and the Bayesian Information Criterion (BIC) (36)), by letting users select the solution that maximizes the likelihood of the trained model given the data. Several drawbacks should be considered, though, as this algorithm assumes the data is structured in a way in which clusters of patients in the feature space follow Gaussian distributions, which is not necessarily the case. Moreover, this approach will always report a solution (there is always a combination of parameters that maximizes the likelihood given the assumptions of the model, regardless of how good the fit to the actual data is). In this study, although a *robustness* analysis is carried out and the reported stability indices across 100 iterations are high, it is unclear if perturbations to the data were applied at all, or if the authors merely re-ran the algorithm on the same dataset. In the latter case, we would recommend taking their stability claims with caution.

Following the method described above, this study reported a five-component solution where clusters seem to differ mainly by disease severity. The authors noted, however, that severity alone did not explain the retrieved components, as (in concordance with their hypothesis) different clusters were enriched in distinct sets of symptoms.

Finally, the authors attempted to interpret the retrieved components using resting-state functional MRI from a subset of the initial subjects (*n* = 251), from which they obtained both dynamic and static functional connectivity networks (dFC and sFC, respectively). While this did not lead to any conclusion for dFC networks, significant results for two of the clusters were found for sFC in the default mode and the frontotemporal networks, both of which are extensively associated with depression in the literature (84, 85).

#### Schizophrenia

The other three studies in this section focused on clustering subjects with Schizophrenia. Having in mind how evident cognitive decay is in patients with the disease (86), Geisler et al. (46) decided in 2015 to search for subtypes on a set of 18 features derived from behavioral and cognitive scores, instead of pure clinical variables. This built on previous research on Schizophrenia subtyping, where it had been reported that clusters based on pure clinical features were longitudinally unstable: psychotic symptoms and disorganization, in particular, are highly variable across time, which causes subjects to change labels often when models are trained using diagnostic systems directly (48).

The dimensionality of the dataset as mentioned above (*n* = 129) was reduced using a linear principal component analysis (PCA), which works by projecting the data into its subsequently orthogonal most prominent modes of variation. A four-cluster solution was later obtained running the K-means algorithm (39) on the first eight components of this reduced space. While a standard pipeline in data science, successfully applied in a plethora of domains, researchers selected both the number of principal components to keep and the number of clusters (as K-means requires the user to define this beforehand) to match previous literature (47), without a concrete analysis of how this selection would affect their solution. In these cases, as was suggested above for Maglanoc et al., there are several pipelines to follow and determine the number of groups present in the data in a systematic way (36). As many already presented unsupervised algorithms, K-means will always report a solution for the given number of clusters, and special care needs to be taken to avoid subtypes that might be overfitting the dataset. Consequently, while the authors were able to interpret their four obtained clusters in terms of their mean feature values, it remains unclear whether this corresponds to the optimal cluster solution in terms of robustness and generalizability.

Once obtained, the clusters' correlates with both structural and task functional MRI [during a blocked working memory paradigm called SIRP (87)] were explored and compared to healthy controls (*n* = 165). This yielded specific patterns of cortical thickness changes in the hippocampus, the lingual gyrus, the occipital face, and Wernicke's areas for different clusters, all previously linked to schizophrenia in the literature (49, 88, 89). Interestingly, task fMRI correlates were found for two of the clusters. One of them, defined by face episodic memory, slowed processing speed, and increased verbal fluency, showed an increased neural activity in the planum temporale [one of the main reported brain areas for language processing (90)]. The other, defined by a deficit in general intellectual function, was found to be correlated with increased neural activity in the parietal operculum and precuneus cortices [both linked to schizophrenia in the literature (91, 92)].

In 2018, Dickinson and colleagues published an article (49) in which they attempted a different approach by clustering data coming from the Positive And Negative Syndrome Score (PANSS), a widely-used standardized schizophrenia-specific symptom scale proposed by Kay et al. in 1987 (88, 89). Using a sample of 549 individuals comprising only diagnosed patients, they attempted unsupervised subtyping using the two-step SPSS clustering algorithm (90, 91), which fits a likelihood-based model to the data in a way that allows the handling of both categorical and continuous variables in the same model. By minimizing the aforementioned Bayesian Information Criterion (BIC) across different numbers of clusters (92), the authors obtained an optimal solution with three components, characterized as deficit (with enduring negative symptoms and diminished emotionality), distress (with high emotionality, anxiety, depression, and stress sensitivity), and low-symptomatic. While the algorithm was run 1,000 times with random reordering of the data, no pipeline with cross-validation (leave out sample) approach was reported. This carries the risk of biasing the *robustness* estimates, as readers cannot know if the reported clusters would hold in an even slightly different dataset.

Meanwhile, a subsample of 182 patients balanced across clusters was exposed to functional MRI scans during a working memory task. Here, their three components showed differential activation of the frontoparietal working memory network, including the right dorsolateral prefrontal (DLPFC) and left parietal cortices, and the left anterior cingulate, all of which had been linked to schizophrenia before (50, 51, 93). The low-symptom group in term showed significantly greater activation in the right DLPFC than the two more symptomatic groups, a healthier pattern mainly linked to working memory and cognitive flexibility (94).

Lastly, a similar approach was followed by Chen et al. in 2020 (56). Using a bigger sample of 1,545 patients diagnosed with schizophrenia, they used Non-Negative Matrix Factorization (NMF) to reduce the dimensionality of patients' PANSS score data. NMF compresses the feature space into a user-defined number of factors by decomposing the data into two non-negative matrices: a basis matrix (called dictionary) with factors as columns, and a factor-loading matrix representing symptomatology of individual patients in the training set (57). Besides, the algorithm imposes an orthonormality constraint that promotes a sparse, more interpretable, representation (95). Using this approach, the extensive PANSS data was reduced to just four values (one per retrieved factor) per individual. These reduced data were then clustered into two components using the fuzzy C-means algorithm (96), which can be thought of as a soft version of the k-means mentioned above, in which each subject is assigned a probability of belonging to each cluster instead of a hard cluster label only. This helps to deal with outliers, usually yielding more robust solutions in real-world data (97).

It is important to highlight here the extensive validation pipeline that this study, in contrast to the previously mentioned in this section, applied in each described step. For dimensionality reduction, we highlight that several standard factor concordance indices (98, 99) were computed for a range of factors across 10,000 runs on random half-splits of the data. Clustering stability was tested by subsampling, bootstrap resampling, and leave-one-site-out replication on a deliberately heterogeneous external sample of 490 patients recruited from nine hospitals across Asia, Europe, and the US. In both steps of the pipeline, the most *robust* solutions of four factors and two clusters were kept.

In addition, the two clusters, when projected on the original PANSS data, were revealed to be mainly representing patients with more prominent positive and negative symptoms respectively. Using functional MRI data derived from a balanced sample of 84 patients, researchers applied a Support Vector Machine [a classification algorithm (100)] to sort subjects in both clusters using functional connectivity features. An overall feature importance analysis of this classifier was used to interpret the components on the functional side, showing the ventromedial frontal cortex, the temporoparietal junction, and the precuneus as the most critical networks whose connectivity differed between clusters. All of these networks have not only been linked to Schizophrenia before but also, in concordance with the authors' interpretation of their clusters, to discriminating between positive and negative symptoms (97–99).

### fMRI Used for Validation of Subtypes Obtained via Unsupervised Learning of Biomarker Data

In this second section, we will discuss three studies (published across four papers) in which the obtaining of biotypes was attempted applying unsupervised learning techniques to sets of biomarkers other than functional MRI itself. This is a particularly important approach, born from the assumption that different biological manifestations of disease can lead to the same phenotypic outcome (as discussed in more detail below).

#### Psychosis

The first study is composed of two articles, published by Clementz et al. and Meda et al. in 2016 (58, 60), on identifying psychosis biotypes. While the first article deals with the obtaining of the biotypes themselves, the second analyses their functional correlates using resting-state functional connectivity.

The term psychosis refers to several pathologies that lead to a deteriorated perception of reality (101). In concordance with what was explained above, the authors claim that different etiologies underlying psychotic symptoms do not necessarily overlap with the available symptom-defined labels (schizophrenia, schizoaffective disorder, and bipolar disorder with psychosis), as symptomatic outcomes may represent the convergence of distinct biological entities. With this in mind, they gathered 1,872 samples from patients diagnosed with any of these diseases (*n* = 711), their first-degree relatives (*n* = 883), and comparable healthy subjects (*n* = 278). The data consisted of biomarker panels comprising neuropsychological markers, cognitive assessment tasks [such as stop signal and saccadic control (101, 102)], and auditory paired stimuli and oddball evoked brain responses assessed by electroencephalography (EEG). Patient data were used for clustering, while relatives and controls served for result validation. Authors further reduced the dimensionality of their dataset by running a Principal Component Analysis (PCA) per modality, selecting the number of components to keep using the elbow in the variance explained curve (92). This yielded a reduced set of 9 features, which were fed into a K-means algorithm from which authors reported a three-component solution. Cluster selection was carried out by maximizing the *gap* statistic (59), which is higher for solutions in which distances between data points within a cluster are consistently smaller than distances between clusters. Cluster robustness to perturbation was assessed *via Jackknife* (103), an approach in which the model is trained as many times as individuals in the dataset, leaving each time a different individual behind.

As hypothesized, cluster assignment did not merely recapitulate the DSM derived labels: they observed that clusters (or biotypes) differed beyond outcome severity, and manifested distinct overall profiles, such as (1) *impaired cognitive control and low sensorimotor response*, (2) *impaired cognitive control but exaggerated sensorimotor response* and (3) *near-normal cognitive and sensorimotor characteristics*. Furthermore, differential cortical thickness of key brain areas was found via voxel-based morphometry (VBM) such as the frontal, cingulate, temporal, and parietal cortices, as well as the basal ganglia and thalamus.

In the follow-up study, individuals in an independent sample (*n* = 1,125) were assigned to the already defined clusters. When comparing patients to relatives and healthy controls, the authors found significantly reduced functional connectivity (both globally and across specific biotypes) in nine networks consistent with previous reports (104–109), and with areas known to be compromised in psychiatric disorders in general, including cognitive control, working memory, attention and introspective thought maintenance. Importantly, all these deficits are claimed to track cognitive control factors more closely, suggesting potential implications for both disease profiling and therapeutic intervention.

The remaining two studies used structural MRI to find disease subtypes and projected their findings into resting-state functional connectivity data afterward.

#### Autism Disorder

The first of the two, published by Chen et al. in 2018 (61), attempts to find Autism Disorder (ASD) subtypes in a sample of 356 diagnosed patients. Taking into account the evidence of atypical neuroanatomy within patients with ASD (110), and the fact that subjects exhibiting different clinical symptoms showed distinct brain structural abnormalities (111), the authors used features extracted from a voxel-based morphometry analysis on structural MRI. Interestingly, the clustering was not performed on these features directly. Instead, researchers computed the *structural difference* between each ASD diagnosed patient and a set of matched healthy controls (*n* = 403), and then applied the aforementioned Non-negative Matrix Factorization algorithm for dimensionality reduction into 60 components representing differences in brain structure between cases and controls. By applying a simple K-means algorithm, authors were able to retrieve a three-component solution. Cluster number selection was carried out by maximizing the silhouette index (62), a statistic that, as many presented already, reflects how concentrated the values of the resulting components are within their respective clusters. While robustness analyses were carried out (by running the algorithm 10 times with random 80% subsets of the data), it is worth mentioning that authors do not report having tested the presence of clusters at all in the data (i.e., number of clusters equals to one).

When validating and interpreting their results, authors first reported differences in disease severity between clusters, as assessed by the Autism Diagnostic Observation Schedule (ADOS) score (112). Besides, when comparing the resting-state functional connectivity networks for each patient in each cluster to healthy controls, they found statistically significant differences in two of the clusters. In both cases, ASD patients showed diminished connectivity in the default mode network, the frontoparietal network, the cingulo-opercular network, the sensory-motor network, and the occipital network, all of which had been linked to autism disorder before (113–117). While more validation studies are needed, this paper provides evidence toward ASD not being a neuroanatomically homogeneous disease.

#### Internalizing Disorders

The last study in this section focused on finding structural subtypes in subjects with internalizing disorders, which are characterized by anxiety, depressive, and somatic symptoms. In this study, Kaczkurkin et al. (63) took a different approach to disease subtyping. Instead of clustering diagnosed patients in a fully unsupervised way, they used a semi-supervised approach called HYDRA (64), which uses the binary case-control labels to find different disease subtypes regarding their difference to controls. This way, the approach is conceptually similar to the paper by Chen et al. cited immediately above, although the difference between cases and controls is not processed directly, but a part of the clustering algorithm.

Thus, using HYDRA in volumetric and cortical thickness data from 1,141 individuals (715 cases and 426 controls), they found a two-cluster solution when maximizing robustness as assessed by the Adjusted Rand index (ARI) during a 10-fold cross-validation scheme (which consists of running the algorithm 10 times, each leaving a different random tenth of the data out). In addition, the functional connectivity of 40 subjects balanced across these two defined categories was obtained in the frequency space (118), which has the advantage of enabling the direct comparison of structural and functional measures using the same atlas (119). The functional measures reflect the average connectivity of a particular region of interest, in this case, delimited by differential structural features. By physically delimiting their functional search by the structural characteristics of their clusters, authors make the assumption that detected changes in connectivity would be directly influenced by the changes in structure, which is a debated concept that was not put directly in place by the studies proposed so far (120, 121). When interpreting the retrieved clusters, researchers reported that one of them was marked by reduced cortical thickness, and showed impaired cognitive performance and higher levels of psychopathology. On the functional side, moreover, this same cluster displayed abnormal connectivity in frontolimbic regions, which is consistent with poorer cognitive performance as reported in the literature (122).

### fMRI Used for Brain Disease Subtyping Directly

The last results subsection will deal with studies in which biotype obtaining was attempted from functional MRI data itself. Eleven articles (ten original studies and a relevant replication) comprising four disorders were included, of which ten relied on resting-state functional or effective connectivity and one in a task-based setting.

#### Schizophrenia and Related Disorders

Starting with Schizophrenia and its related disorders, in 2014, Du et al. (65) published an article in which the distinction between Schizophrenia (SZ) itself, psychotic bipolar disorder (BD), Schizoaffective disorder with depressive episodes (SADD), and Schizoaffective disorder with manic episodes (SADM), all of which share overlapping sets of symptoms and genetic landscapes (123, 124), was recapitulated using functional connectivity data clustering (65). This built upon the fact that, for all four, differences in functional connectivity between cases and controls had been reported, which had significantly raised the interest in delineating the functional implications of these diseases over the last few years (125, 126). In addition, the authors attempted to shed light on the controversy on whether SAD is an entity in itself or the manifestation of some degree of interaction between SZ and BD (127). To process their data, they used Independent Component Analysis [ICA, a standard technique for obtaining correlated brain networks (128)] to yield functional connectivity data from a sample of 93 subjects, balanced across all diagnosis categories (including healthy controls).

While pioneering the use of unsupervised learning on resting-state data, this paper illustrates one of the major issues with feature selection in clustering (129). Given that, *a priori*, this study deals with a high number of brain connectivity features and a relatively low number of samples, the authors proceeded to reduce the dimensionality of their data. However, instead of using an unbiased technique such as the aforementioned PCA or NMF, the authors fitted classifiers to discriminate between the five classes in a supervised manner and retained the most informative features. They accomplished this by using a standard technique called Recursive Feature Elimination (130), which measures how impactful the removal of certain features (in this case brain networks) is for a classifier to distinguish between entities. Even though they arrive at a nearly perfect 5-cluster solution (recapitulating their original four diseases and healthy controls), the problem arises from the fact that the features they used were selected to overfit the classification they already had, which makes the clustering trivial. Furthermore, we believe a warning of caution should be raised on the final conclusion of the study, which uses the distances between retrieved clusters (which had been artificially maximized) as evidence to support the hypothesis of Schizoaffective disorder being an independent etiological entity.

Another article that dealt with dissecting the mechanistic underpinnings of Schizophrenia and its potential subtypes was published by Brodersen et al. in (66, 86). In this proof-of-concept study, the authors employed Dynamic Causal Modeling (DCM) to retrieve a *directed connectivity* model from a balanced sample of 83 subjects, including diagnosed patients and healthy controls. While they present a plethora of demonstrative approaches in their study, here we will only discuss their unsupervised clustering, which implicated two separate pipelines: first, authors were able to recapitulate the classification between cases and controls with relatively high accuracy (~71%) using only clustering on the whole sample. Second, and arguably more interesting to this review, the exclusion of the healthy controls led to a clustering solution of three components (*n* = 41), which seemed to differ mainly by symptom severity, as assessed by the aforementioned PANSS scale.

To reach these solutions, researchers applied a Variational Bayesian Gaussian Mixture Model, a variant of the likelihood approach presented above for Maglanoc et al. which runs automatic cluster selection by estimating how many components of a *prior* distribution are present in the data. While this algorithm is appealing for small studies, finite Gaussian Mixture Models as the ones presented above are still preferred in many settings, given their lower computational complexity and their fewer associated implicit biases (131).

While a mere pilot study where the main goal was to explore and define a working pipeline, the authors use these results as an argument to defend the exclusion of healthy controls in the unsupervised learning procedure, as the likelihood of the already-known binary factor is high (the variance in the data might in many cases be dominated by the disease-control distinction). However, we believe that a follow-up study should review if these premises hold in a bigger sample, and assess how generalizable and *robust* the solutions are using internal and external validation, as was highlighted throughout the review.

A different approach was taken by Yang et al. in 2014 (69) when investigating early-onset Schizophrenia (EOS) in a small sample of 52 individuals, balanced across medication-naïve diagnosed patients and age and gender-matched healthy controls. The authors used a pipeline called gRACIAR (generalized ranking and averaging independent component analysis by reproducibility) (132) to obtain both subject-specific functional connectivity networks (via Independent Component Analysis) and a meta graph concerning intersubject similarity within each functional connectivity network. Using a maximal-clique community detection algorithm, a clustering procedure that, unlike all presented above, works on a graph level (70), researchers reached a clustering solution for each of the retrieved networks. Importantly, the similarity thresholds for drawing the edges of the mentioned metagraph were selected based on the average solution robustness to permutation tests during cross-validation.

While no communities (the equivalent to clusters in graph theory) were retrieved for the majority of the explored networks, two of them yielded interesting results. First, a component involving the precuneus-angular gyri (PCU-AG, associated with the default mode network), was detected to significantly recapitulate the case-control separation, which suggested a novel association between these functional connectivity features and EOS. Second, a network involving bilateral superior temporal gyri and bilateral inferior frontal gyri yielded a solution enriched in diagnosed patients, which seemed to recapitulate the difference between positive and negative symptoms (as assessed for example with the PANSS scale).

While the retrieved clusters revealed little new about the disease substructure across subjects as a whole, this approach allowed for the discovery of associations within networks that had not been previously reported. Furthermore, the question of whether more interesting clustering solutions from an EOS functional subtyping point of view could be retrieved with a bigger sample size remains.

#### Major Depression Disorder

Shifting to Major Depression Disorder, Drysdale et al. (73) reported in 2017 a four-cluster solution from resting-state functional connectivity data, using a training sample of 220 diagnosed patients. For dimensionality reduction, they applied an algorithm called Canonical Correlation Analysis (CCA), which, instead of selecting the most prominent modes of variation in one dataset as many of the approaches presented above (such as PCA or NMF), takes two data modalities and returns a space in which the correlation between them is maximized (74). In this case, the authors decided to apply it to a combination of the functional connectivity data, coming from fMRI, and the subjects' HAM-D scores [one of the most common self-assessed symptom scales for MDD (133)]. This type of analysis can be particularly useful for high-dimensional data where the major components of variability are not expected to be related to the problem at hand. In this case, the assumption is that there might be other sources of variance, such as sex, age, brain size, etc. that might overshadow the implications in functional connectivity of potential MDD subtypes. This way, a transformation of the biological data that correlates with psychiatric symptomatology is reported, making it likely that the downstream clustering will focus on relevant connectivity features.

After applying this pipeline, the authors retained the first two canonical variates (which one could see as analogous to principal components in this context) obtained from CCA, which they interpreted as anhedonia and anxiety-related by checking correlation with individual symptoms. Using a Hierarchical Agglomerative Clustering approach, they reached a four-component solution by maximizing the so-called Calinski-Harabasz (CH) index, a statistic similar to the Silhouette presented before, that measures how similar a datum is to its own cluster compared to others (75). Drysdale et al. (73), These components lay on each of four quadrants defined by two axes, interpreted by the authors as anhedonia and anxiety-related. Interestingly, both clusters associated with high anxiety profiles were linked to abnormal connectivity patterns in the frontal amygdala [fear-related behavior and reappraisal of negative emotional stimuli (134)] and abnormal hyper-connectivity in the reward system was especially pronounced in anhedonia-related clusters.

Aside from providing innovative methods and focusing thoroughly on the generalizability of the achieved results, this article incentivized active discussion in the field, especially after a replication attempt published by Dinga et al. in 2019 (78). When failing to reproduce the original results after applying nearly the same pipeline on a smaller independent cohort of 187 diagnosed individuals, the authors highlighted potential statistical weaknesses in the original study.

First, they claimed there was a statistical bias in the reported CCA results. While the original article alleged that both canonical variates' correlation with symptoms were statistically higher than random, the problem arose from a two-step process that Drysdale et al. applied. From the functional connectivity matrices obtained from fMRI, they selected voxels whose activations were most correlated with symptoms and *then* employed only those features on the CCA analysis. Furthermore, the first selection step was ignored in the statistical tests they ran [based on Wilk's lambda statistic, typically used for this purpose across the literature (135)], and permutation testing in the replication study showed that the significant correlations between symptoms and connectivity faded away when taking into account this pre-selection of voxels. This made it seem likely that the original procedure was selecting noise in the direction of the hypothesis. Moreover, CCA is known to be prone to overfitting (reporting correlates between modalities that are much stronger than they would be on an independent dataset). While Drysdale et al. did not evaluate this problem directly, 10-fold cross-validation in the replication revealed it was a significant issue, raising even more caution toward the reported CCA factors.

Lastly, even when Drysdale et al. assessed internal and external validation of their findings (by measuring cluster stability across 10,000 random splits of the data, and using an independent multisite dataset, respectively), they did not test the null hypothesis of whether there was an inherent clustering structure in the data against the possibility of a continuum (a single, unimodal distribution). When testing this using previously described methods (79), they found no significant evidence supporting a clustering structure. In summary, while some details of the proceedings were not the same as in the original, this article shows how important thorough statistical testing (which considers every step involved in all relevant pipelines) is in these complex scenarios of multiple data integration and how crucial replication attempts are. While Dinga et al. do not discard the possibility of subtypes of depression that are identifiable at a functional level, they raise a warning of caution about the lack of strong evidence supporting it, and call for more extensive methodological evaluation in an incipient field.

In another pioneering study, Price et al. were the first to our knowledge, in 2017, to use *effective* connectivity to build *directed* resting-state networks using causal modeling for MDD subtyping (67). The pipeline employed (called Group Iterative Multiple Model Estimation, or GIMME) has been shown to reliably recover both the presence and direction of connectivity among brain regions per individual in simulations (136). Using a sample of 80 diagnosed patients with Major Depression, the authors built a similarity matrix between model parameters among individuals, which they thresholded into a graph. Here, they reached a two-component solution via a clustering algorithm called Walktrap (68), which works under the assumption that short-distance random walks in a graph tend to stay in the same community. It is a *hard-clustering* algorithm, in the sense that a label is assigned to every patient, without any associated metric reflecting how confident the model is in each case. Furthermore, even though this approach arrives automatically to an optimal number of clusters in the data (according, that is, to its own definition of what a cluster is), neither *cluster robustness* analyses nor estimates of how generalizable their solution might be on external data were provided in this study. Besides, although innovative in their methodologies based on causal, directed connectivity, their method is computationally demanding, which limits the resolution of the brain activation networks they can use when compared to methods based on functional connectivity.

Mapping back their retrieved components to functional connectivity, authors observed that one of the two retrieved groups showed a connectivity pattern across DMN nodes concordant with what was previously reported on average depressed patients (85). The other subgroup showed, however, a different pattern in this region, with increased dorsal anterior cingulate-driven connectivity paths. This group also had significantly higher comorbidity with an anxiety disorder and highly recurrent depression, which led to a poorer outcome of the disorder. Interestingly, altered connectivity in anterior cingulate regions (belonging to the DMN) has been more recently linked to persistent sadness and higher recurrence rates (137), which goes in concordance with these results.

In summary, while the employed sample size is small and further validation is highly encouraged, this study illustrates how graph theory and causal modeling can be used together to shed light on the mechanistic heterogeneity behind major depression in particular and brain disorders in general.

As previously mentioned, an issue that researchers often encounter when applying clustering algorithms to a problem is that, even when a relevant structure is present in the data, it can be overshadowed by variance factors that are ultimately unrelated to the problem. The most typical ways of dealing with this issue are to control for *known* confounders in our models, such as age or sex (138), to directly model and remove their variability (139), or to transform data in a way that maximizes its correlation with a highly informative variable [as previously presented for CCA and symptom scores (73)]. Tokuda et al. (77), however, introduced a custom algorithm that tackled the problem in a very different way: they arrived at multiple *solutions* (or *views*) simultaneously, which corresponded to *different modes of variation* in the data. This way, they could select *a posteriori* if any of them was actually related to subtypes of disease (in this case MDD) and still extract potentially useful insights about their samples from the rest. Furthermore, each of these *views* attempts to solve a so-called co-clustering problem, in which both subjects and features are grouped. This means that individual solutions won't be forced to adopt all the available information, ideally using only those features that are relevant to them. Moreover, the algorithm they propose is capable of simultaneously dealing with categorical and continuous variables, allowing researchers to integrate resting-state functional connectivity data with other data domains, such as BDI questionnaires, biomarker panels, genetics, and methylation data from a preselected set of related genes.

When applying this approach to a sample of 134 subjects, balanced across diagnosed patients and healthy controls, the authors reached a five-component solution after selecting the view that maximized the Cohen's D coefficient [a statistic that measures effect size (140)] between the two groups. Interestingly, two clusters were mainly composed of controls, whereas the other three included diagnosed patients almost exclusively. Moreover, these three MDD-related reported clusters were observed to differ significantly by functional connectivity between the Angular Gyrus (and other already reported brain areas in default mode network), child abuse trauma scale scores (CATS), and selective serotonin reuptake inhibitor treatment outcomes (although all of these were used directly for clustering). Cluster stability (*robustness*) was tested via leave-one-out cross-validation (similar to the aforementioned Jackknife) on the whole pipeline, but no external validation was accounted for. While the employed sample size is relatively small, and the results demand replication in independent datasets, this article proposes an innovative and assertive approach with a high potential for integrating distinct data domains.

#### Attention Deficit Hyperactivity Disorder

Another article that relied on effective directed connectivity, and applied the aforementioned GIMME algorithm, was published in 2014 by Gates et al. (120). In this study, the authors attempted to cluster a sample containing also 80 individuals, balanced across subjects diagnosed with attention-deficit/hyperactivity disorder (ADHD) and healthy controls.

After following a pipeline nearly identical to the one presented above for Price et al. (67), the study reported a solution with five components, two of which were almost exclusively composed of ADHD-diagnosed patients. First, researchers generated a network in which subjects were connected when the similarity between their directed connectivity patterns is high (*how* high was determined by measuring cluster *robustness* under a cross-validation scheme). For clustering, they used a *hard* community detection similar to the Walktrap mentioned above, which partitions the network into non-overlapping communities by maximizing a metric called *modularity* (that compares the number of edges within a community to those that connect it to other partitions) (141).

The obtained subgroups were reported to be highly distinguishable by their differential connectivity in regions such as the dorsolateral prefrontal and frontal cortices, the intraparietal sulcus, and the inferior parietal lobule, all of which had been previously linked to ADHD in the literature (142). Furthermore, the inclusion of healthy controls at clustering time, and their presence even in clusters highly dominated by diagnosed subjects, made it interesting to consider that the reported brain findings may reflect liability for ADHD in subgroups that are biologically at risk. Rather than ADHD *per se*, the controls in these groups may represent individuals at risk for ADHD who had sufficient protective factors in their development (or their genome) to avoid exhibiting the syndrome. Although inconclusive, this article, as many in this review, provides evidence toward the presence of biological subtypes in yet another psychiatric disease, which can be recovered at a functional level.

Using a functional connectivity pipeline on a sample of 106 children (aged 7–12 years), including both diagnosed patients and controls, Costa Dias et al. also attempted to find data-driven subtypes of ADHD in their article published in 2015 (71). One of the main highlights of this study is that, in order to reduce the original dimensionality of their functional connectivity data obtained from resting-state fMRI, the authors restricted the problem physically, by including only brain areas that had been previously reported as related to the disease. To accomplish this, they built a mask using a meta-analytic tool called NeuroSynth (143), which yielded a set of brain regions that highly overlapped with the reward system. This constitutes a well-studied connectivity hub, which interacts with other brain networks to promote decision-making, and has been extensively shown to be altered in ADHD (72). From the resulting connectivity features, researchers extracted a meta-correlation matrix that was thresholded into a graph, and applied the same network-based modularity-based algorithm mentioned for the previous paper by Gates et al. Using this approach, authors arrived at a three-cluster solution, whose stability was assessed by randomly perturbing the aforementioned network 20 times. *Robustness* was then assessed using a metric called variation of information (VOI), which measures how much information differs between the two sets of community assignments, and varies from 0 (identical) to 1 (completely dissimilar) (144).

This article reported that connections between the nucleus accumbens and the default mode network were atypical in ADHD across all the three subgroups, a finding that was previously reported by the same group (145). The authors, however, arrived at this conclusion by comparing diagnosed patients to controls in each of the three reported communities. Furthermore, one of the main drawbacks of this study was that it seems to have failed to recapitulate disease manifestation along with the clustering solution, making it seem likely that the factors of variance captured by the applied methods do not correspond to the disease axis. Specifically, this would mean that the most prominent mode of variation in connectivity across the reward system does not correspond, at least in their sample, to the manifestation of the disease.

Having this issue in mind, Lin et al. published in 2018 (76) what constituted the last attempt to date (to our knowledge) to find biotypes of ADHD using resting-state fMRI. In this article, the authors used a sample of 80 diagnosed subjects and 123 matched healthy controls, to extract networks that, across the entire dataset, were differentially activated between both groups. This approach yielded differential activations predominantly between the default-mode, cingulo-opercular and subcortical networks, all of which had been previously reported as related to ADHD as a whole (62, 146). They then attempted to use this data to specifically contrast what they called a *dimensional biotype* (i.e. heterogeneity arises from variation over a continuum of the same entity) against a *categorical biotype* (different pathological entities explain the observed variability in the data, which converge in similar symptomatology).

To further deal with unwanted modes of variation, they applied a variant of the aforementioned canonical correlation analysis (CCA) to bring into the picture the maximum correlates between their differential functional connectivity and symptomatic scores. From this analysis, they were able to retrieve just one significant mode of covariation between both data modalities, which was interpreted as the first piece of evidence supporting a *dimensional biotype*. Moreover, they attempted to cluster the data using two distance-based clustering algorithms: K-means and spectral clustering, both of which yielded an optimal solution supporting the absence of discrete biotypes in the data. This was concluded after maximizing the *robustness* of the obtained results, as measured by already presented metrics, such as the Jaccard and silhouette indices, and the gap statistic (59).

While the overall conclusion of this paper supports the idea of ADHD being a single biological entity, we believe the presented evidence is inconclusive, and that a few concerns should be raised. For starters, while the sample size is said to be large enough to deal with the applied clustering algorithms given their number of features (34), this may not consider the complex feature selection/extraction that was employed. It is possible that even though it is technically possible to apply these algorithms to a sample this small, not enough variation is captured in their original dataset to represent with confidence potential categorical biotypes that might exist in the population. Second, the first step in their feature extraction pipeline involved the usage of only those networks that were differentially activated between cases and controls overall. While this can be useful, as mentioned, to dissect modes of variation that are related to the problem at hand, it also carries the risk of leaving behind brain connectivity features that might differ significantly between controls and particular subsets of patients (the biotypes). In other words, filtering by overall variation might bias the data toward features that correspond to a dimensional biotype.

#### Consequences of Early Trauma

The last study presented in this section, published by Sellnow et al. in 2020 (80), delves into the functional consequences of extreme stress in early childhood. Early stress events (such as interpersonal violence -IPV- or severe trauma) are one of the major causes of subsequent psychopathology, and no systematic studies had attempted to disentangle their underlying heterogeneity in neither the type nor the magnitude of their consequences (147).

To tackle this problem, the authors used a sample of 114 adolescent girls (aged 11–17), from which they obtained functional MRI data during an emotion processing task in a blocked design. After filtering the voxels of interest using a meta-analytic mask obtained from the aforementioned NeuroSynth (related to emotion processing), the GLM-first order coefficients were concatenated and clustered across individuals using the K-means algorithm. After selecting the best model using the already presented elbow method on the cluster validity index [a statistic that, like many introduced before, compares intra-cluster to inter-cluster density (34)], they reached a three-component solution, shown robust *via* leave-one-out cross-validation.

At a functional level, the retrieved clusters were distinguishable by engagement of the medial prefrontal cortex, the anterior insula, and the hippocampus, all involved in emotion processing (which is not surprising, given brain features had been filtered using a meta-analytic mask using this criterion). Interestingly, when analyzing the relationship between each cluster and external measures of interpersonal violence (IPV) and internalizing symptoms, the authors managed to report a ‘healthier' component, in which exposure to violence had been lower, and two clusters with high symptom severity, that seemed to differ on the presence or absence of sexual assault. Furthermore, IPV exposed a negative correlation with symptom reduction over Trauma-Focused Cognitive Behavioral Therapy (TFCBT), which led the authors to suggest the feasibility of their methodology to predict treatment outcomes based on functional information.

As many studies presented in this review, this last one attempts to set the ground for further exploration of an incipient field. One concern about their methods, though, is the high dimensionality of the used data. Having retained 3,970 voxels after filtering, and using a GLM with blocks of 4 different tasks, each of the 114 individuals ended up represented by 15,880 values. Although one could argue that these features are far from independent (after all, they represent the task-importance of voxels that are contiguous in space), this extremely high relationship between dimensions and samples can lead to overfitting, severely decreasing the generalizability of the models to external samples. This problem, often referred to as the “*curse of dimensionality*”, is a very common drawback to many machine learning models which usually justifies the need for dimensionality reduction (148).

## Discussion

### One Problem, Multiple Approaches: Top-Down, Bottom-Up, and Polytopic Learning

Throughout this article, we gave an overview of the most recent attempts to subtype psychiatric disease in a data-driven manner. Across 20 studies, we illustrated how functional MRI, arguably the most relevant proxy of brain function to date, was applied to both validation and interpretation of clusters retrieved with other techniques, and as part of the clustering pipelines themselves.

Furthermore, these categories are encapsulated within two broad ways of dealing with subtyping in data-driven medicine, which we would like to call *top-down* and *bottom-up* approaches. The former corresponds to what was presented in the first section of this review: the use of data comprehending the clinical and behavioral manifestation of disease, and the attempt to validate the retrieved components relying on the elemental biology. The latter is the opposite (second and third sections): clusters are defined based on the biology and validated at a clinical/behavioral level.

In this context, the first section of the results illustrated how unsupervised learning could be used to detect subgroups in psychiatric symptom data (Table 2). As briefly discussed in the introduction, this approach is likely to yield disease symptomatic states rather than biological entities, given that different sets of symptoms do not necessarily reflect distinct etiologies. Symptomatic profiles, moreover, are sensitive to treatment and environmental perturbations, among others. This may reflect in patients changing cluster assignments during the course of their disease, making the usage of this type of solution hard for diagnostic and prognostic models. Along the same lines, however, this type of approach can be very helpful to better evaluate the state of a patient at a given time, which constitutes an arguably different but equally relevant problem than the one we are presenting here.

The second and third sets of articles (Tables 3, 4) focused on a *bottom-up* approach. When clustering biomarkers, the assumption is that the data capture the manifestation of the disorder at a lower level (hence *bottom*), yielding results that are potentially closer to uncovering pathological origins. This is particularly relevant when considering that distinct biological entities (which can have distinct optimal treatments) can converge to an equivalent symptomatic profile. For example, studies have shown how different genetic alterations that produced different structural consequences led to the same set of autistic-like behavioral traits in mice (49).

**Table 3:** Retrieved articles in which fMRI was used to interpret biomarker-based clusters.

**Publication year**	**Reference**	**Pathology**	**Data used for clustering**	**Sample size (symptoms)**	**Data used for validation**	**Sample size (fMRI)**	**Dimensionality reduction**	**Clustering**	**Cluster number selection**	**Robustness testing**	**Healthy Controls included**	**Testing against continuum**	**Reported subtypes**	**featured brain areas/ *networks***
2016	Clementz et al. (58)	Psychosis	biomarker panels	1,872	–	–	PCA (57)	K-means (36)	GAP Statistic (59)	Jackknife (34)	No	Yes	3	–
2016	Meda et al. (60)	Psychosis	–	–	rsfMRI	1,125	–	–	–	–	No	–	–	*cuneus-occipital, fronto-parietal, cerebellar-occipital, default mode, bilateral temporo-parietal, fronto-parietal*
2018	Chen et al. (61)	ASD	sfMRI (VBM)	356	rsfMRI	356	NMF (57)	K-means (36)	Silhouette index (62)	random splitting (34)	Yes, Indirectly	No	3	*default mode, frontoparietal, cingulo-opercular, sensory-motor, occipita*l
2019	Kaczkurkin et al. (63)	MDD	sfMRI (cortical thickness)	1,141	rsfMRI	40	raw features	HYDRA (64)	Adjusted Rand Index (62)	cross-validation (34)	Yes	No	3	frontal regions, right amygdala, right hippocampus

**Table 4:** Retrieved articles in which fMRI was used to cluster subjects into biotypes.

**Publication year**	**Reference**	**Pathology**	**Data used for clustering**	**Sample size (fMRI)**	**Data used for validation**	**Sample size (validation)**	**Dimensionality reduction**	**Clustering**	**Cluster number selection**	**Robustness testing**	**Healthy Controls included**	**Testing against continuum**	**Reported subtypes**	**featured brain areas/*networks***
2014	Du et al. (65)	SCZ	functional connectivity	93	–	–	Recursive feature elimination	K-means, HAC (36)	based on previous knowledge	algorithm reinitialization - no data perturbation	Yes	No	5	frontal, parietal, precuneus, cingulate, supplementary motor, cerebellar, insular, and supramarginal cortices
2014	Brodersen et al. (66)	SCZ	effective connectivity	41	PANSS symptom scale	41	raw features - weights of DCM models	VBGMM (36)	automatic	not reported	Yes	Yes	3	visual–parietal– prefrontal working-memory network
2014	Gates et al. (67)	ADHD	effective connectivity	80	–	–	raw features - weights of DCM models	Walktrap (68)	automatic	network permutation	Yes	Yes	5	dorsolateral prefrontal and frontal cortices, intraparietal sulcus, inferior parietal lobule
2014	Yang et al. (69)	SCZ	functional connectivity	51	PANSS symptom scale	51	raw functional connectivity features	maximal clique (70)	automatic	cross-validation	Yes	Yes	2	precuneus-angular gyri
2015	Costa Dias et al. (71)	ADHD	functional connectivity	106	behavioral measures	101	meta-analytic masking (NeuroSynth) (72)	Walktrap (68)	automatic	random perturbation	Yes	Yes	3	nucleus accumbens, default mode network
2017	Drysdale et al. (73)	MDD	functional connectivity	220	HAM-D scores (symptoms)	1188	CCA (functional connectivity - symptoms) (74)	HAC (36)	CH index (75)	random splitting (34) external validation in independent samples	No	No	4	limbic and frontostriatal networks
2017	Price et al. (67)	MDD	effective connectivity	80	clinical data	80	raw features - weights of DCM models	Walktrap (68)	automatic	network permutation	No	Yes	2	default mode network, dorsal anterior cingulate nodes
2018	Lin et al. (76)	ADHD	functional connectivity	80	behavioral measures	80	CCA (functional connectivity - symptoms) (74)	K-means, spectral clustering (36)	jaccard, silhouette, gap	-	No	Yes	1	default-mode, cingulo-opercular and subcortical networks
2018	Tokuda et al. (77)	MDD	functional connectivity - biomarker data	134	CATS score, response to medication	134	raw features	custom multi-view co-clustering	automatic	cross-validation	Yes	Yes	5	default mode network, angular gyrus node
2019	Dinga et al. (78)	MDD	functional connectivity	187	HAM-D scores (symptoms)	187	CCA (functional connectivity - symptoms) (74)	HAC (36)	CH - silhouette indices (75)	cross-validation	No	Yes (79)	1	–
2020	Sellnow et al. (80)	IPV	emotion processing task fMRI	114	behavioral measures	114	meta-analytic masking (NeuroSynth) (72)	K-means (36)	cluster validity index (75)	cross-validation	No	Yes	3	medial prefrontal cortex, the anterior insula, hippocampus

Among the methodologies overviewed in the second section, structural MRI clustering (encompassing 2/20 studies) and its projection into functional data deserve special mention, as several studies have shown that psychiatric disorders have structural implications (149). While diseases such as Autism or Schizophrenia are generally recognized as neurodevelopmental disorders with brain structure being affected, there are inconsistencies regarding the regional specificity of the neuroanatomical findings (149), making the importance of structural subtyping apparent. Furthermore, the search for functional correlates of these subtype-specific functional alterations relies on assuming that an altered structure may lead to an altered function. By combining the two data types, it is possible to test this hypothesis, retrieving multiple domains affected by the disorder that may be coupled with a non-trivial causal relationship.

Delving into the third and last set of articles (Table 4), we want to highlight that fMRI is the most direct measure of brain function we have to date. Although not ideal, it constitutes arguably the best available proxy for the biological manifestation of brain disease. This carries the potential to shed light on mechanistic biotypes reflecting distinct pathological entities that overlap at higher levels. Both task and resting-state approaches have been explored, although the vast majority (10/11) of studies opted for the latter given its more straightforward implementation and potentially broader conclusions and generalizability (39).

It shouldn't go unnoticed that many studies (4/20, all in the third section) integrate both symptom and biological data in several clever ways. This set of approaches, which lies arguably in the interface of the *top-down* and *bottom-up* presented above, belong to what has been called *polytopic learning* (39). By either combining both kinds of data for clustering directly (77) or relying on multimodal transformations such as CCA (73, 76, 78), researchers seek to bridge the gap between origin and manifestation of disease, in search of what have been described as *endophenotypes* (150). We think this has an incredible potential *a priori*, as illustrated by the many proofs of principle in this review. However, it implies extending the dimensionality of the datasets and, up to now, limiting sample sizes for reaching strong conclusions. However, the future, in this regard, looks promising.

### Deep Validation of Retrieved Biotypes

As previously mentioned throughout this review, the interest in disease classification (and sub-classification) is far from new. Aside from expanding basic knowledge, taxonomy as a whole serves the purpose of recognizing distinct, stable entities that may be treated differently, which can directly lead to improving people's lives. In this sense, unsupervised learning has the potential to help researchers discover these entities given the right data, models, and experimental designs. Whereas, in other medical subfields clinical application is more tangible (151, 152), psychiatry carries the weight of a perfect storm: on top of currently relying on fuzzy symptom-defined labels, relevant functional data collection is expensive, making sample sizes to date (as seen throughout this review) limitingly low.

This is far from discouraging though, as the field is still at an early stage where methodological exploration seems to be the rule. It does highlight, however, the importance of thoroughly validating the retrieved results on several equally relevant dimensions, in what some authors have called *deep validation* (153) of biotypes. This concept encompasses three main axes: (1) replication of clustering solutions in independent data, to assess methodological generalizability (2) application of a retrieved clustering solution to new independent data (without reclustering), to gauge whether the new assignments correspond to clinically meaningful outcomes, and (3) extension of clustering solutions defined in a cross-sectional manner to a longitudinal setting, to determine if baseline components yield, for example, differential trajectories of disease progression.

When exploring how these three concepts were touched upon across the systematically retrieved literature, we found that the first deep validation component, which goes in line with the notion of generalizability discussed above, was the most explored throughout the available corpus. Even in mostly proof-of-principle settings, 15 out of 20 studies engaged in robustness and generalizability analyses (Tables 2–4). Most of them, however, yielded intra-sample reports (by partitioning one available dataset instead of using truly external data) which can lead to inflated generalizability estimates (37). While nearly all studies (17/20, Tables 2–4) undertook an interpretation of their solution using clinically relevant measures, only four of them (56, 58, 60, 73) attempted to report generalizability across multiple data collection sites, using truly external data. Moreover, only two (60, 73) attempted the application of the retrieved biotypes to an independent sample (second deep validation component). Among these, interestingly, Drysdale et al. (73) reported significant differences in outcome after the patients were treated with transcranial magnetic stimulation (TCMS), which constitutes a perfect illustration of the potential utility of biotyping as mentioned above. Furthermore, only one of the retrieved studies to date has engaged in longitudinal validation (56). This one, unsurprisingly, used symptom data to cluster given its easier collection across time.

While the landscape we found is far from ideal, the lack of thorough, standard deep validation pipelines reflects, in our opinion, the scarcity of relevant available data in such an early stage of the field rather than deep methodological flaws.

### Methodological Heterogeneity: Should We Strive for Standardization?

Whereas most of the papers followed overall similar formulas (data preprocessing, select relevant features or reduce the dimensionality of the dataset, and cluster the available samples), we observed vast methodological variability in every step along the way.

As differences in dimensionality reduction approaches were discussed throughout the corpus of the article, in this section we will focus on the coarse classification of clustering algorithms provided in Table 1. Here, we observed that the majority (12/20) of the studies employed distance-based methods. An equal number of papers applied algorithms that work at the graph level and model-based approaches (4/20 each). Whereas we think that, given the fuzzy nature of the available labels and how important the measure of uncertainty in the medical setting is (154), model-based approaches may be the most intuitive way to go, we found the preference for simpler, computationally cheaper models understandable given the incipient state of the field. It is refreshing to see, however, that even at this incipient stage several customized algorithms, designed specifically with the problem of biotyping in mind, have been presented (77, 120).

A topic that deserves special attention is the inclusion of healthy controls. Of the 20 retrieved studies, 6 decided to treat healthy controls as any other sample, one utilized them indirectly by clustering differences between matched subjects (61), and one treated them as a normative reference for semi-supervised learning (63) (Tables 2–4). As made evident by the variability in the literature [the strongest retrieved example being the three retrieved papers on ADHD (67, 71, 76)], this is not a closed topic and valid arguments on both sides exist. On the one hand, their inclusion may lead to an obvious first mode of variation in the data. This can lead to clustering algorithms finding the division between cases and controls as the dominant solution, which would yield no new insights on disease functioning. On the other hand, however, if sufficient data modalities are available and subtypes are prominent enough, the inclusion of healthy controls makes sense as a way to represent the true nature of the population. Finding clusters enriched in healthy controls can thus be interpreted as a mild form of validation. Moreover, individuals in disease-enriched clusters who were not diagnosed might correspond to early stages of disease, or be a consequence of the presence of protective factors that may lead to further investigation.

Finally, the available variability opens an important question: Should we strive for methodological standardization to remove potential dependencies of the results on the employed metrics? For now, we do not think so. First, because at such an early stage it is important to develop proofs of principle that work on the intended data, and no single algorithm has yet shown sufficient advantage. Second, because we think that the biggest source of inconsistency across the literature today comes from the data itself, not the algorithms employed. If the retrieved clusters are strong enough, researchers should be able to retrieve overlapping solutions regardless of the clustering methodology. A word of caution here is, though, that as different algorithms make different assumptions, we should make sure they are met in the datasets we use. We strongly believe that the future of the field lies in concerted efforts to acquire more data.

### Overview of the Field and Where to Go Next

Psychiatric disease subtyping is at the moment at an incipient, exploratory stage. While much remains to be answered and no irrefutable evidence of functionally relevant subtypes was presented, all the cited proofs of principle introduced valid, potential ways to move the field forward once the current limitations are overcome.

Aside from the algorithmic heterogeneity mentioned above, the number of different data modalities to choose from should not go unnoticed. Although we focused mainly on the functional aspect of disease and functional MRI as the most promising way of measuring it, psychiatric diseases can manifest at many levels, which can be captured across several different axes that can be included in any clustering effort. Other imaging techniques, such as the already discussed structural MRI or diffusion tensor imaging (DTI, useful for measuring white matter consistency across the brain) have also been used to detect relevant subtypes (38). Furthermore, the genetic components of many of these subtypes should not be ignored. As the dimensionality of this data is extremely high (millions of genetic variants per subject) and individual polymorphism contributions are generally small, however, genetic data is rarely useful for unsupervised learning. Supervised approaches, however, which aim to classify individuals among already defined labels, have shown more success (37). Lastly, in addition to questionnaires and more traditional clinical datasets, a data modality that gained momentum over the last few years is digitomics (electronic health records, mobile sensor data) (37, 155, 156). We think that, given sufficient sample sizes, the future lies in multimodal integration and, as stated previously, *polytopic learning*.

Furthermore, fMRI results may depend on external unmeasured factors, which often results in low signal-to-noise ratios and poor test-retest reproducibility (157). A relevant consequence of these limitations is that the sample sizes needed to capture the modes of variation in line with psychiatric subtypes are, to date, limitingly high. This demands concerted efforts to increase data collection, which are fortunately being accounted for, with new multi-site data collection consortia starting to collect functional data for psychiatric disease machine learning (158–160).

Although its details are out of the scope of this review, something that should not go unnoticed is that any effort in acquiring knowledge that aims to be transferable to the clinic needs to comply with standards of *fairness* (161). In this case, this reflects the need for models to be thoroughly tested across samples representative of the entire population to which they ought to be applied. As functional MRI hardware is expensive, bias in data collection toward richer societies is a significant risk (161). Fortunately, new technological advancements, such as portable MRI (162), also make the future look brighter in this regard: by reducing costs and the required infrastructure, solutions like this one can help bridge this gap and facilitate data collection across the world.

Moreover, the aforementioned limitations of fMRI, even if robust subtypes are available, make it a relatively poor clinical tool (163). This means that, even if robust subtypes at the functional level are detected, their application in clinical workflows might need to rely on technologies other than functional MRI. This could be achieved for example by training supervised classifier models to recognize these functionally defined subtypes based on data from other modalities, such as combinations of genetics, digitomics, and imaging (37).

Finally, all the retrieved studies aimed to find subtypes within already defined broad categories. Although several *transdiagnostic* efforts using other, readily available data modalities exist (38, 164), none to date have, to the best of our knowledge, applied functional MRI as part of their pipelines. As new, larger datasets are made available, the goal of shedding light on the functional aspects of trans diagnosis becomes reachable.

## Conclusions

As mentioned throughout this article, further data-driven stratification of psychiatric diseases can help dissect the vast heterogeneity present in the field today. An improved diagnosis, presumably based on biological mechanisms that precede symptom manifestation, is not only a goal in itself but also key for improving disease prognosis and direct personalized treatment. Functional MRI, and brain connectivity, in particular, is positioned as the best tool to date to acquire insights into brain function, and the interest in using it for uncovering sub-entities of brain disease remains high. The presented results are however mixed, and much remains to be done in terms of increasing sample sizes, standardizing data collection, and providing models with strong assessments of generalizability and fairness, crucial for a future translation of any model to the clinic (13).

## Data Availability Statement

Publicly available datasets were analyzed in this study. This data can be found here: https://zenodo.org/record/3923919#X0ei3NP7TmE.

## Author Contributions

LM has organized the PRISMA workflow, gathered and scrutinized information, and written the entire manuscript draft. RP has contributed to the inclusion/exclusion criteria, commented on the initial draft, suggested initial revisions to the manuscript's structure, and re-written several parts of the original draft. BP has greatly contributed to figure design. BP, NK, and BM-M have contributed with PRISMA assessment, proofreading of the document, and suggestions based on their expertise in the field. All authors contributed to the manuscript and approved the submitted version.

## Funding

This project has received funding from the European Union's Horizon 2020 research and innovation programme under the Marie Skłodowska-Curie grant agreement No. 813533 and the Commitment grant No. 01ZX1904A (Modellierung von Komorbiditäts-Prozessen durch integratives, maschinelles Transfer-Lernen für psychiatrische Erkrankungen).

## Conflict of Interest

The authors declare that the research was conducted in the absence of any commercial or financial relationships that could be construed as a potential conflict of interest.

## Publisher's Note

All claims expressed in this article are solely those of the authors and do not necessarily represent those of their affiliated organizations, or those of the publisher, the editors and the reviewers. Any product that may be evaluated in this article, or claim that may be made by its manufacturer, is not guaranteed or endorsed by the publisher.

## Footnotes

^1^Both direct inclusion of healthy controls or indirect referencing to normative samples (i.e. by clustering differences between diagnosed patients and matched controls) were considered.
